# Potential role of anaerobic plant-associated bacteria in naphthenic acid degradation

**DOI:** 10.1128/aem.00410-26

**Published:** 2026-04-27

**Authors:** Simon Morvan, Sara Correa-García, Marie-Josée Bergeron, Kaitlyn Trepanier, Ian J. Vander Meulen, Dilini M. Atugala, Julien Tremblay, Jason M. E. Ahad, John V. Headley, Lisa M. Gieg, Dani Degenhardt, Christine Martineau, Étienne Yergeau

**Affiliations:** 1 Centre Armand-Frappier Santé Biotechnologie, Institut National de Recherche Scientifique, Laval, Canada; 2 Laurentian Forestry Centre, Canadian Forest Service, Natural Resources Canada 98661 , Québec, Canada; 3 Northern Forestry Centre, Canadian Forest Service, Natural Resources Canada 98660 , Edmonton, Canada; 4 National Hydrology Research Centre, Environment and Climate Change Canada 197341 , Saskatoon, Canada; 5 Department of Civil, Geological and Environmental Engineering, College of Engineering, University of Saskatchewan 7235, Saskatoon, Canada; 6 Department of Biological Sciences, University of Calgary 2129, Calgary, Canada; 7 Geological Survey of Canada, Natural Resources Canada 492807 , Québec, Canada; Shanghai Jiao Tong University, Shanghai, China

**Keywords:** constructed wetlands, naphthenic acids, biodegradation, anaerobic bacteria, *Carex aquatilis*

## Abstract

Plant-associated microorganisms can break down many contaminants, including naphthenic acid fraction compounds (NAFCs)—one of the toxic contaminants in oil sands process-affected water (OSPW). One way to take advantage of these processes is to use constructed wetland treatment systems (CWTSs), where aquatic plants work in coordination with microorganisms to remediate OSPW. However, the dynamics of the microbial communities and the potential contribution of distinct bacteria and fungi inhabiting sediments, rhizosphere, and water that comprise these systems remain poorly understood. To address this, we planted water sedge (*Carex aquatilis*) in mesocosm-scale wetland systems and compared it with unplanted controls. Naphthenic acid fraction compound (NAFC) concentrations and microbial community dynamics among various mesocosm compartments were monitored using mass spectrometry and high-throughput sequencing of the bacterial-archaeal 16S rRNA gene and the fungal ITS2 region. *Carex*-planted mesocosms reduced NAFC concentrations more rapidly than the unplanted controls, which coincided with shifts in abundance for both bacterial and fungal communities, especially in the *Carex* rhizosphere. The strongest relationships between microbial taxa and the decrease in NAFC concentrations were for anaerobic bacteria, suggesting a prominent role for these bacteria in NAFC degradation in CWTS. This study highlights potential microbial targets for improving CWTS efficiency for NAFC detoxification during OSPW remediation.

**IMPORTANCE:**

Naphthenic acid fraction compounds (NAFCs) represent one of the most toxic and persistent contaminants in oil sands process-affected water (OSPW), posing a significant environmental challenge. As oil sands operations continue, large volumes of OSPW have accumulated, requiring effective treatment before any potential environmental discharge. Our study explores bioremediation through constructed wetland systems as a sustainable approach to reduce NAFC concentrations. By uncovering the microbial dynamics involved in NAFC degradation, our research provides critical insights into the identification of microbial communities that can enhance the remediation process. This manuscript contributes to advancing the scientific foundation needed to develop effective, biological solutions for OSPW treatment.

## INTRODUCTION

During phytoremediation, plants and associated microorganisms work together to degrade or sequester environmental contaminants (1–3). Constructed wetland treatment systems (CWTSs) use phytoremediation to treat large volumes of contaminated water, such as oil sands process-affected water (OSPW) generated during bitumen extraction (4–7). Engineered wetlands offer a sustainable, *in situ,* and cost-effective approach for wastewater phytoremediation (8, 9). Field and pilot-scale studies have demonstrated the ability of CWTSs to reduce contaminant concentrations (4, 10). Even though plants can accumulate and detoxify or segregate pollutants in their tissues (11), microorganisms are the primary degraders of organic contaminants during phytoremediation (1). Plants are particularly efficient at stimulating microbial degraders, as many plant secondary metabolites are chemically analogous to organic contaminants (12). However, the key microbial players in CWTS for the remediation of OSPW remain poorly understood (13), impairing the optimization of the system and the removal of a key contaminant, naphthenic acids.

Naphthenic acid fraction compounds (NAFCs) constitute one of the main contaminants of concern in OSPW. Their structure allows them to disrupt cellular membranes and make them highly toxic, especially among freshwater communities (14). NAFC-degrading microorganisms have been reported and include aerobic and anaerobic bacteria, such as *Pseudomonas, Acinetobacter*, *Aromatoleum, Geobacter,* and *Pseudoalteromonas* (15–21), as well as microalgae (22–24). However, the interactions between these degraders and aquatic plants, central to the efficiency of CWTS, are not well known.

Previous work from our group used CWTS planted with *Scirpus microcarpus* and *Triglochin maritima*, but with sediments of low nutritional quality, which hindered plant growth and did not support a significant microbial community, obscuring the impact of plant presence on NAFC degradation (25). Nonetheless, we found potential NAFC degraders, such as *Rhodoferax*, *Ensifer*, *Trichormus, Leptolyngbya,* and the *Trebouxiophyceae* green algae family in the plant environment. Here, we compare the microbial community in *Carex*-planted vs unplanted CWTS mesocosms fed with OSPW. In this experiment, *Carex* presence was shown to significantly increase the degradation of NAFC as compared with the unplanted mesocosms (7). We hypothesized that this effect of plant on the degradation of NAFC would be mirrored in shifts in microbial communities. We therefore used amplicon sequencing to study the microbial communities associated with plants, sediments, and water throughout the 84 days of this greenhouse mesocosm experiment and linked them to observed differences in NAFC degradation.

## RESULTS

**Fig 1:**
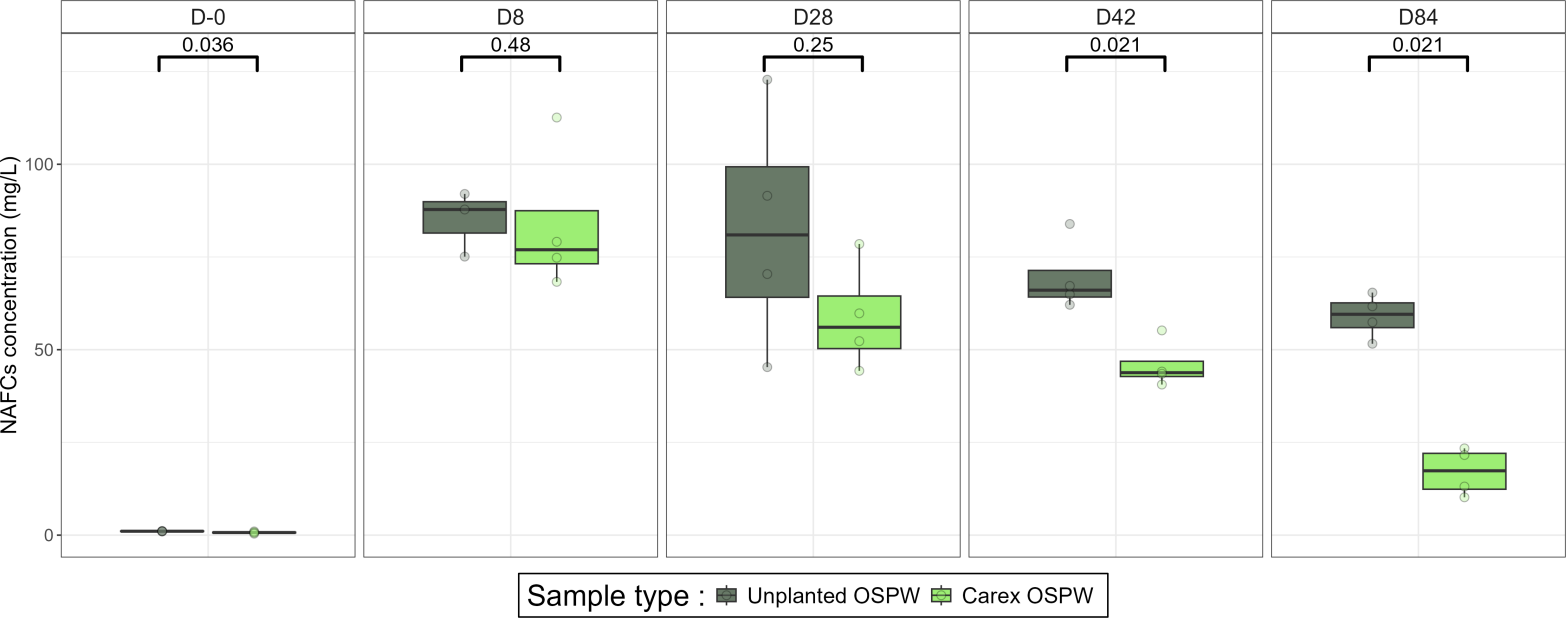
Comparison of NA concentrations in the water column between unplanted and Carex-planted mesocosms. Statistical significance was assessed using a Kruskal-Wallis test, followed by a post-hoc Dunn’s test with Bonferroni *P* value corrections (data from reference 7). Values below 0.05 indicate significant differences between unplanted and *Carex* mesocosms for that particular date. D-0, D8, D28, D42, and D84 correspond to the number of days following addition of OSPW to mesocosms.

Trepanier et al. (7) showed that the concentration of total NAFCs in the water decreased significantly within the *Carex*-planted mesocosms compared with unplanted mesocosms (Dunn’s test, Bonferroni correction—Fig. 1). Given these results, we compared the microbial communities present within the *Carex*-planted mesocosms to the ones present in the unplanted mesocosms.

### Shifts in alpha and beta diversity

Fungal and bacterial alpha diversity indices in planted and unplanted sediments and *Carex* rhizosphere were not significantly different from each other (Fig. S1 and S2). Time of sampling had little effect on the alpha diversity indices regardless of the sample types. In sediments from *Carex*-planted mesocosms, fungal richness declined gradually from D-0 to D84 (significant difference between D-0 and D84, Dunn’s test, *P* value = 0.0034, Bonferroni correction; Fig. S3), while in the *Carex* rhizosphere, bacterial richness increased progressively from D-0 to D42 (significant difference between D-0 and D42, Dunn’s test, *P* value= 0.0066, Bonferroni correction; Fig. S4). In the water column, the presence of plants resulted in a significant diversity increase in bacterial ASVs compared with the water column of unplanted mesocosms, with 219 ± 43 ASVs in the planted OSPW and 172 ± 59 ASVs in the unplanted OSPW (Dunn’s test, *P* value < 0.05, Bonferroni correction; Fig. S5). With respect to sample timing, we obtained an overall trend of increased alpha diversity indices with time in the bacterial data set (Fig. S7).

**Fig 2:**
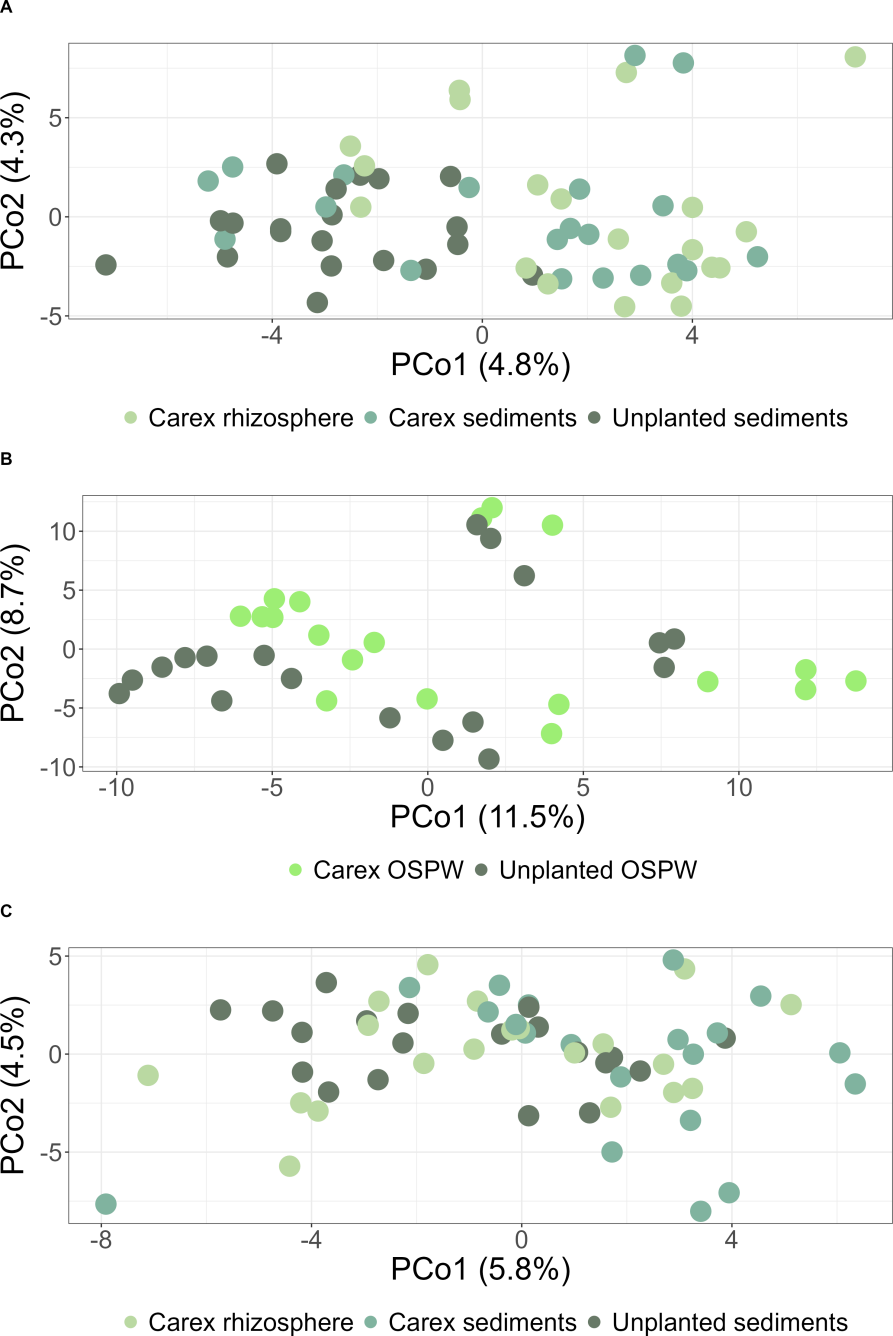
Ordination presenting the beta diversity of the microbial communities based on robust Aitchison distance matrices and principal coordinate analysis (PCoA). (**A**) Bacterial data set in solid phase (sediments and rhizosphere); (**B**) Bacterial data set in water column; (**C**) Fungal data set in solid phase (sediments and rhizosphere).

In both bacterial and fungal data sets, community composition associated with root samples was significantly different from the one associated with the rest of the samples (Fig. S9 and S10). To enhance focus on differences between samples of *Carex* rhizosphere and sediments from planted and unplanted mesocosms, roots were excluded from the PERMANOVA and ordination plot. Although it is not visible in the first two dimensions of the ordinations (Fig. 2) because of the overwhelming influence of time, significant differences were found in both bacterial and fungal community composition among the three sample types (*Carex* rhizosphere, sediments from planted and unplanted mesocosm) when controlling for temporal variation (Bacteria: F = 1.46, R² = 0.05, *P* value = 0.001; Fungi: F = 1.29, R² = 0.04, *P* value = 0.001, Fig. 2A and C). Pairwise PERMANOVA results revealed that bacterial and fungal communities inhabiting sediments from unplanted mesocosms were significantly different from the ones inhabiting sediments from planted mesocosms and *Carex* rhizosphere (*P* < 0.05, Bonferroni correction). However, we found no significant differences in diversity between sediments and rhizosphere in the planted mesocosms (*P* value > 0.05, Bonferroni correction). Based on this lack of difference, subsequent comparative analyses were limited to *Carex* rhizosphere vs. sediments from unplanted mesocosms.

Time of sampling also had a significant effect on bacterial and fungal community composition (bacteria: F = 1.47, R² = 0.10, *P* value = 0.001; fungi: F = 1.40, R² = 0.09, *P* value = 0.001). A pairwise PERMANOVA test revealed that fungal community composition varied significantly between sampling dates, except for the D-0 vs D8 and D28 vs D42 comparisons. Bacterial community changes were gradual as the D-0 vs D8, D8 vs D28, and D28 vs D42 comparisons were not significant (*P* value > 0.05, Bonferroni correction).

For the bacterial communities inhabiting the water column, a significant difference was also observed between the *Carex*-planted and unplanted mesocosms (F = 1.68, R² = 0.05, *P* value = 0.001) (Fig. 2B). Compared with the analysis of sediments and rhizosphere, time of sampling explained a higher proportion of the variation in water community composition (F = 2.69, R² = 0.25, *P* value = 0.001). Except for the D28 vs D42 comparison, water bacterial communities for each sampling date were different from the others (*P* value < 0.05, Bonferroni correction).

### Shifts at the class level

**Fig 3:**
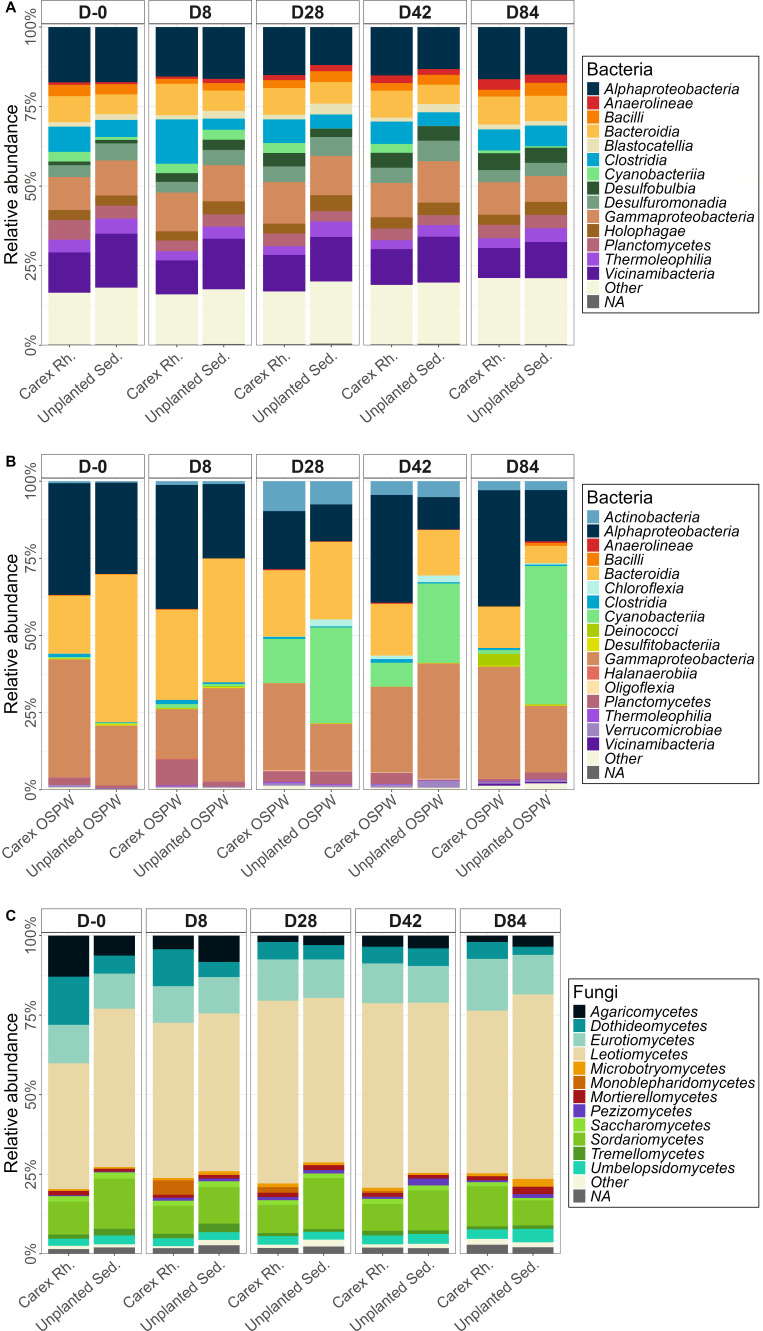
Relative abundances of most abundant taxonomy classes for each sample type and time combination. The remaining classes were pooled in the “Other” category. (**A**) Bacteria in sediments from unplanted mesocosms and *Carex* rhizosphere. (**B**) Bacteria in water. (**C**) Fungi in sediments from unplanted mesocosms and *Carex* rhizosphere.

At the class level, differences were subtle when comparing the bacterial community of *Carex* rhizosphere to the one of sediments from the unplanted mesocosms (Fig. 3A). The three dominant classes in each sample type were *Alphaproteobacteria*, *Vicinamibacteria,* and *Gammaproteobacteria*. Sediment communities had a higher abundance in *Vicinamibacteria* (14.5% ± 1.4%) than in *Carex* rhizosphere samples (11.1% ± 1.2%). Conversely, *Carex* rhizosphere samples contained more *Alphaproteobacteria* sequences (15.9% ± 1.0%) compared with the sediments from unplanted mesocosms (14.7% ± 2.2%). *Clostridia* abundances were higher in the *Carex* rhizosphere (8.6% ± 3.0%) compared with the sediments from unplanted mesocosms (4.8% ± 1.2%). Additionally, in both the rhizosphere and sediments, there was a progressive rise in relative abundances of *Desulfobulbia* starting from D8 and continuing to the end of the experiment in both the *Carex* rhizosphere and sediments from unplanted mesocosms.

Bacterial communities inhabiting the water column were different from those of the sediments as they were dominated by four classes*—Alphaproteobacteria*, *Bacteroidia*, *Cyanobacteria*, and *Gammaproteobacteria*—regardless of mesocosm type (Fig. 3B). One noticeable difference was the higher proportion of *Alphaproteobacteria* sequences in water samples from *Carex*-planted mesocosms (33.3% ± 11.4% versus 17.5% ± 9.0%), in line with observations made in the *Carex* rhizosphere. A temporal trend of decreasing relative abundance of *Bacteroidia* and increasing relative abundance of *Cyanobacteria* was observed in unplanted OSPW. At D-0, *Bacteroidia* accounted for 48.5% ± 11.8% of the bacterial community in water from unplanted mesocosms. Meanwhile, at this same time point, *Cyanobacteria* represented less than 1% of the sequences. At the final sampling date (D84), *Bacteroidia* sequence relative abundance was clearly reduced (6.0% ± 5.0%), while *Cyanobacteria* dominated the community with 44.8% ± 24.7 %.

With respect to fungal communities, samples of *Carex* rhizosphere and sediments from the unplanted mesocosms were relatively similar (Fig. 3C). The *Leotiomycetes* dominated the community (*Carex* rhizosphere: 51.3% ± 7.6%; unplanted sediments: 52.8% ± 3.5%), followed by *Eurotiomycetes* (*Carex* rhizosphere: 13.1% ± 1.9%; unplanted sediments: 11.7% ± 5.5%), and *Sordariomycetes* (*Carex* rhizosphere: 9.8% ± 1.7%; unplanted sediments: 12.8% ± 3.4%).

### Shifts at the ASV and family levels

To better understand the bacterial community variation between *Carex* rhizosphere and unplanted sediments, we computed differential abundance analyses at the ASV level (Fig. S11). The results showed a significant increase of several ASVs in *Carex* rhizosphere after the addition of 100% OSPW in mesocosms (D8 and following dates). These ASVs (some of which were enriched at multiple sampling dates) included three *Hungateiclostridiaceae* (ASVs 47, 90, and 103), two *Desulfurobacterium* (ASVs 48 and 189), and one *Dechloromonas* (ASV 4).

In the water column, differential abundant ASVs were not stably enriched over time (Fig. S12). Before introducing OSPW in the mesocosms, water from *Carex*-planted mesocosms was enriched in several *Chitinophagaceae* (ASVs 28, 72, 8, and 151) and *Sphingomanadaceae* (ASVs 19, 204, and 53). Later in the experiment, water samples from *Carex*-planted mesocosms were enriched, among others, in two *Chitinophagaceae* (ASV 9*—Sediminibacterium* and ASV 274*—Terrimonas*) and two *Comamonadaceae* (ASV 7*—Limnohabitans* and ASV 409—Unknown genus).

For the fungal community, several ASVs were significantly more abundant in the *Carex* rhizosphere across the experiment (Fig. S13). These ASVs belonged to different genera (ASV 33: *Calophoma;* ASV 193: *Penicillium*), including two members of the Helotiales order (ASV 173: *Oidiodendron* and ASV 21: *Leptodontidium*).

We also used indicator species analyses at the family level and found several significant bacterial families, with *Hungateiclostridiaceae*, *Clostridiaceae*, Spirochaetaceae, *Rhodanobacteraceae*, *Rubritaleaceae*, and *Caloramatoraceae* as indicators of the *Carex* rhizosphere samples, and *Gaiellaceae* and *Nitrospiraceae* as indicators of the unplanted sediments (FDR adjusted *P*-value < 0.05). For fungi, we did not find any indicator families or genera in the *Carex* rhizosphere.

### Correlation with NAFC concentration

**Fig 4:**
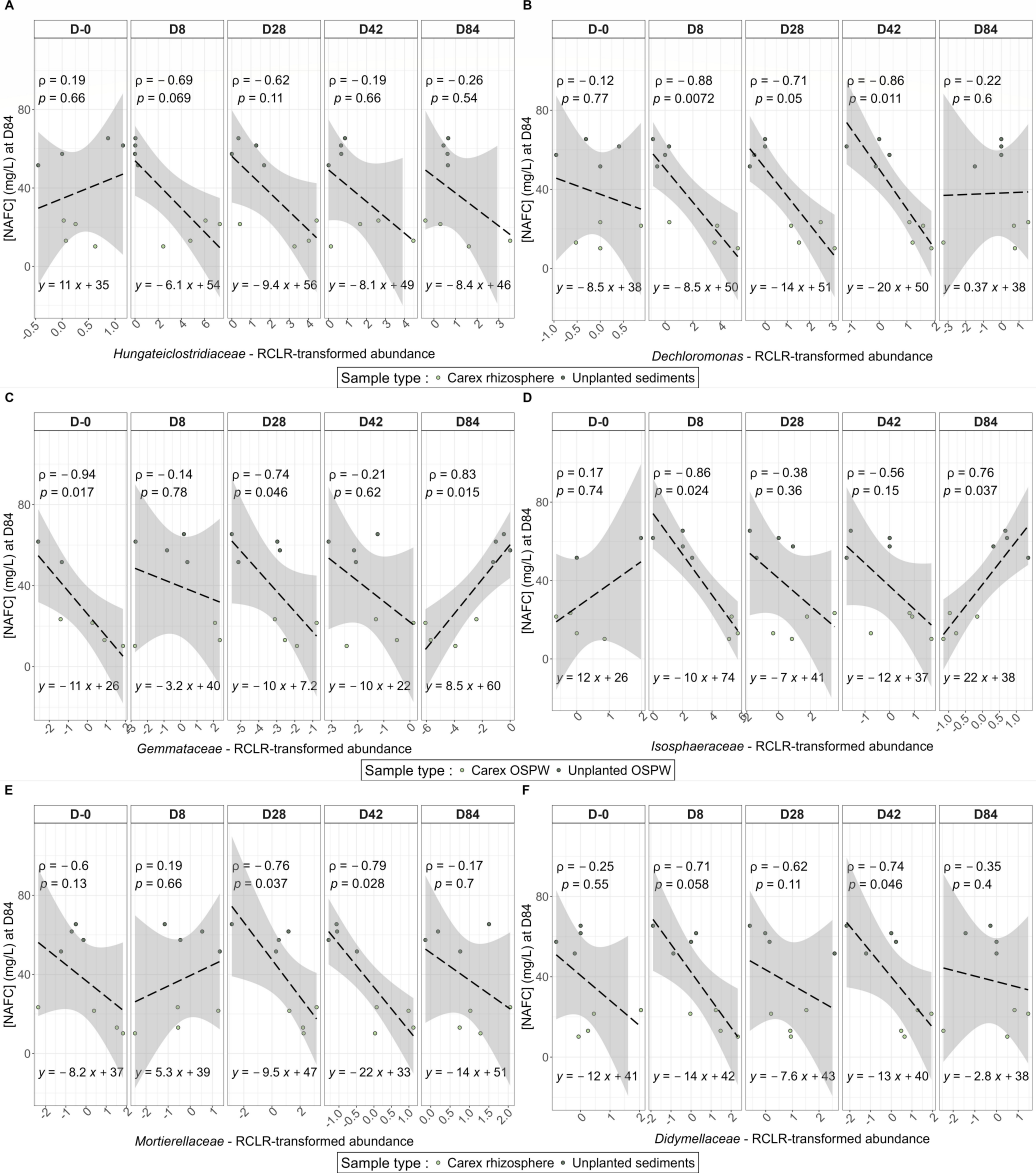
Spearman correlation between RCLR transformed abundance of taxa at each sampling date with the final NA concentration. The Spearman rho and *P* value, as well as the linear regression equation, are annotated within each plot. (**A**) *Hungateiclostridiaceae* in *Carex* rhizosphere and unplanted sediments. (**B**) *Dechloromonas* in *Carex* rhizosphere and unplanted sediments. (**C**) *Gemmataceae* in *Carex* OSPW and unplanted OSPW. (**D**) *Isosphaeraceae* in *Carex* OSPW and unplanted OSPW. (**E**) *Mortierellaceae* in *Carex* rhizosphere and unplanted sediments. (**F**) *Didymellaceae* in *Carex* rhizosphere and unplanted sediments.

Then, for each time point separately, we looked at which families were negatively correlated to the final NAFC concentrations. For the bacteria, *Hungateiclostridiaceae* were negatively correlated throughout the experiment, though it was only nearly significant at D8 (statistical information is provided on each plot; Fig. 4A). A similar trend was observed for *Dechloromonas*, with significant negative correlations at D8, D28, and D42 (Fig. 4B). In the water column, we found significant negative correlations for *Gemmataceae* at D-0 and D28, and a significant positive correlation at D84 (Fig. 4C), whereas *Isosphaeraceae* was negatively correlated at D8 and positively correlated at D84 (Fig. 4D). For fungi, we observed a strong negative correlation between NAFC concentration at D84 and the *Mortierellaceae* relative abundance at D28 and D42 (Fig. 4E) as well as with the *Didymellaceae* relative abundance at D8 and D42 (Fig. 4F).

**Fig 5:**
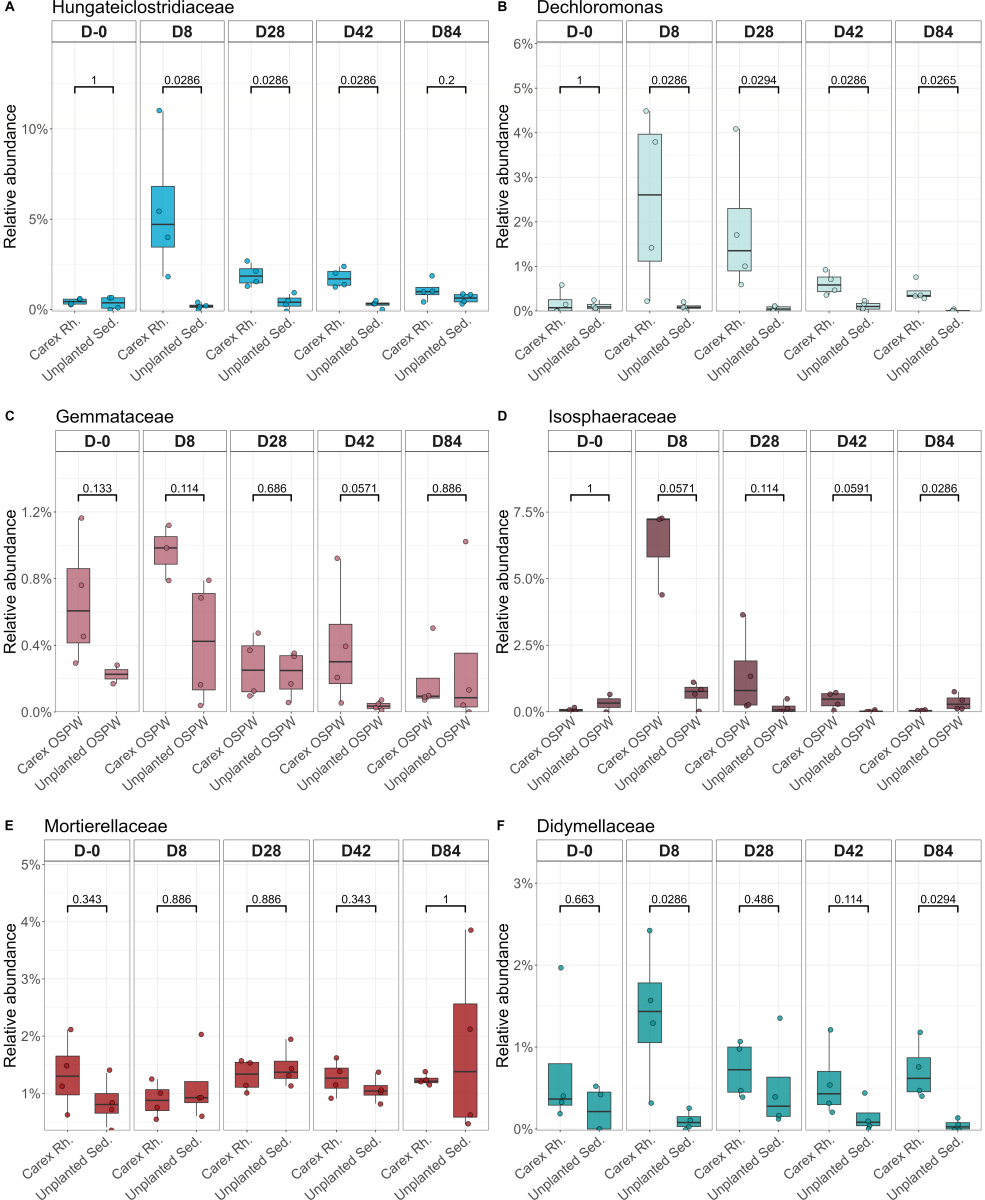
Relative abundance comparison between planted and unplanted mesocosm for specific taxa. These taxa were identified in the Spearman correlation between the taxon’s RCLR transformed abundance at each sampling date with the final NA concentration. (**A**) *Hungateiclostridiaceae* in *Carex* rhizosphere and unplanted sediments. (**B**) *Dechloromonas* in *Carex* rhizosphere and unplanted sediments. (**C**) Gemmataceae in *Carex* OSPW and unplanted OSPW. (**D**) Isosphaeraceae in *Carex* OSPW and unplanted OSPW. (**E**) Mortierellaceae in *Carex* rhizosphere and unplanted sediments. (**F**) Didymellaceae in *Carex* rhizosphere and unplanted sediments. Statistical significance was assessed using a Wilcoxon test.

We then looked at the relative abundance of these families at the different time points in the rhizosphere of *Carex* vs unplanted sediments (Fig. 5). In most cases, we found significant or nearly significant (0.10 < *P* < 0.05) differences between the rhizosphere and unplanted sediments in the relative abundance of the families for at least one time point (Fig. 5). For instance, at D8, 28, and 42, the *Hungateiclostridiaceae* were significantly more abundant in the rhizosphere of *Carex* as compared with the unplanted sediments (Fig. 5A), whereas the *Dechloromonas* were significantly more abundant at D8, 28, 42, and 84 (Fig. 5B).

### Shifts following exposure to OSPW

**Fig 6:**
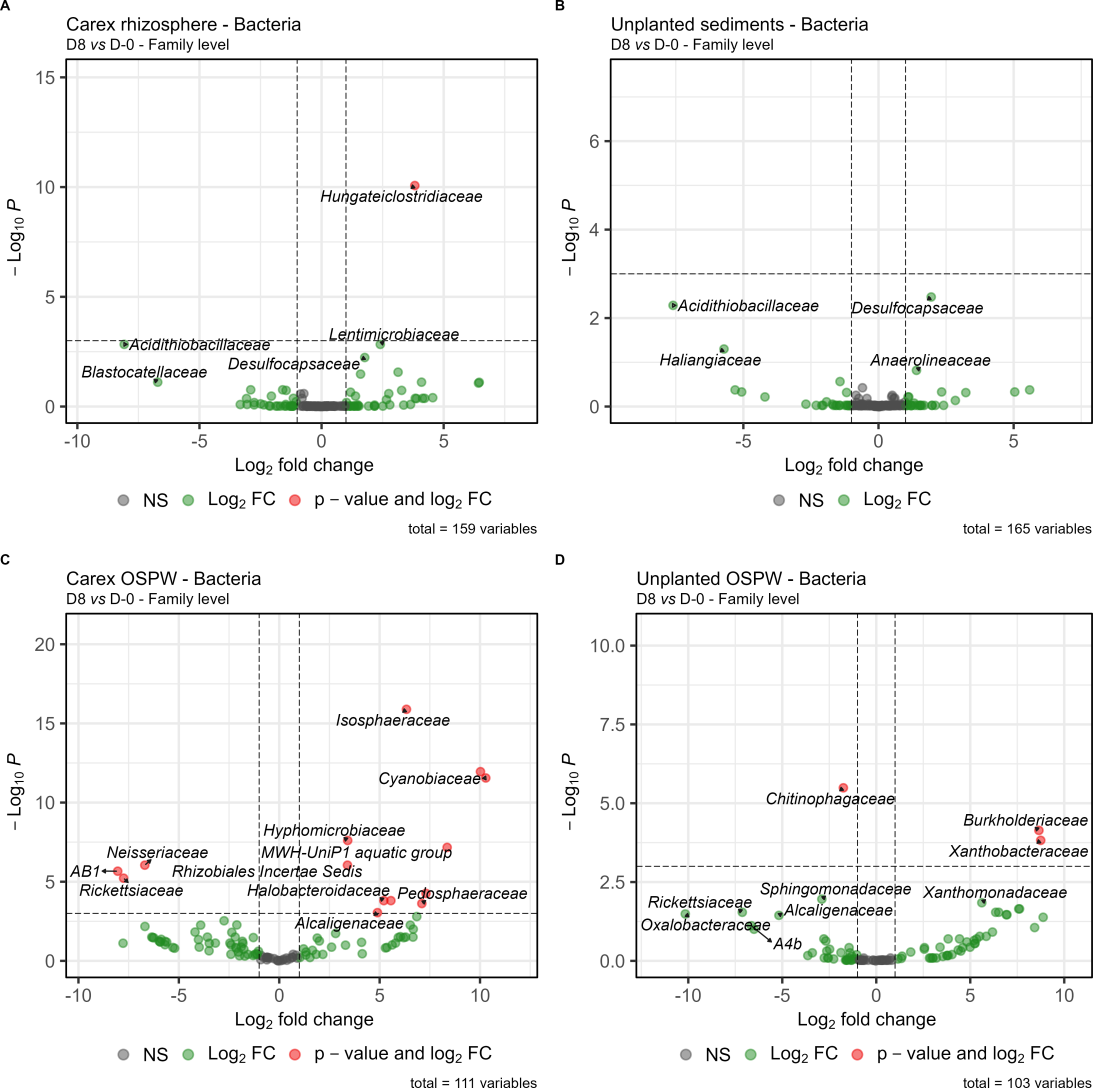
Microbial community changed at the family level after 100% OSPW addition. (**A**) Bacterial community in *Carex* rhizosphere samples. (**B**) Bacterial community in unplanted sediment samples. (**C**) Bacterial community in *Carex* water samples. (**D**) Bacterial community in unplanted water samples.

Finally, we measured the change in microbial community when exposed to OSPW by comparing D8 samples (post-OSPW exposure) to D-0 samples (pre-OSPW exposure) for each sample type (Fig. 6). In the *Carex* rhizosphere samples, *Hungateiclostridiaceae* was the only family enriched at D8 (Fig. 6A). We did not find any significant changes for communities in unplanted sediments (Fig. 6B). In the water column, the presence of *Carex* is also linked with a more pronounced bacterial community change upon the addition of 100% OSPW, compared to the unplanted mesocosms (Fig. 6C and D). Several families (e.g., *Pedosphaeraceae*, *Hyphomicrobiaceae*, *Cyanobiaceae*, *Halobacteroidaceae*) were enriched at D8 in the water column of *Carex* mesocosms, while this was only the case for two families (*Burkholderiaceae* and *Xanthobacteraceae*) in the unplanted mesocosms (Fig. 6C and D). For fungi, we did not observe any significant change in relative abundance following exposure to OSPW (both at class and family levels).

## DISCUSSION

Here, we compared the microbial communities of CWTS mesocosm planted with *Carex aquatilis* or not, that were exposed to OSPW. We had already shown that the presence of *Carex* reduced more rapidly the concentration of NAFC as compared to the unplanted mesocosms (7). We had hypothesized that these differences would be mirrored in shifts in the microbial communities associated with the plant. We can confirm this hypothesis as we found higher bacterial alpha diversity in the circulating water of the planted mesocosms and significant differences in the general and specific microbial communities between the two mesocosms. As phytoremediation efficiency is driven by plant-bacteria partnerships (1, 3), the shifts in microbial communities observed here likely explain the effect of plants on the degradation of organic pollutants, as previously reported (26, 27).

Even though it was not the case in the sediments, we found a higher bacterial alpha diversity in the water column of the planted mesocosm. As there is generally a positive link between diversity and the functional capabilities of the community (28), this difference could partly explain the difference in degradation capability of the planted and unplanted mesocosms. Also, this higher diversity in the water column could have also offered more possibilities for recruitment in the rhizosphere, potentially explaining the differences in the rhizosphere/sediment communities between the planted and unplanted mesocosms. Some of the differences in the water microbial community composition could be related to the differences in degradation capacity. For instance, *Sediminibacterium* and *Terrimonas* (ASVs enriched in the *Carex* water column) were previously reported as potential polycyclic aromatic hydrocarbon (PAH) degraders (29, 30). Similarly, members of the *Comamonadaceae*, including *Limnohabitans* (also enriched in the *Carex* water column)*,* often contain several genes involved in aromatic and alkane degradation genes (31, 32) and are regularly found in oil sands tailings (21, 25).

The plant plays a crucial role in phytoremediation (33–35). Their root systems harbor a variety of active microbes that are primed to degrade root exudates, some of which are complex carbon structures analogous to petrochemical hydrocarbons (1). Consequently, even in the absence of pollutants, rhizospheres are enriched in microorganisms that can degrade organic contaminants (36). Here also, we found that many of the taxa enriched in the planted mesocosms contained members previously reported to degrade organic contaminants. For instance, the fungal family *Didymellaceae* contains strains efficient in anthracene biodegradation (37), whereas *Mortierella* species were also suggested to degrade PAH (38, 39). Both *Didymellaceae* and *Mortierellaceae* were also negatively correlated to the final NAFC concentration. An ASV from the *Calophoma* genus (Didymellaceae) was more abundant in the rhizosphere of *Carex*, and this genus was previously found in oil sands tailings (40). Similarly, the clostridial family *Hungateiclostridiaceae* was negatively correlated with final NAFC concentration, and the family as a whole and various representative ASVs were enriched in the planted sediments. This family dominated the active bacterial community in anoxic biofilters used to treat OSPW (41), among other *Clostridia* taxa. *Clostridia* are regularly dominant in anaerobic enrichments of naphthalene, pure NAFCs, or OSPW (41–45). We also found negative correlations between *Dechloromonas* abundance and the final NAFC concentrations. This genus was also an indicator of the planted mesocosms and has previously been identified as capable of anaerobic degradation of several monoaromatic hydrocarbons (46, 47). Additionally, the *Rhodocyclaceae* bacterial family (which includes *Dechloromonas*) was correlated with NAFCs degradation in an anoxic-aerobic membrane bioreactor treatment system (48). *Dechloromonas* was also the most abundant bacterial genus found in “biotraps” amended with ^13^C-labeled 1-adamantanecarboxylic acid (a model NAFC) and placed in a wetland nearby an oil sands tailings pond (49). While there was no evidence for *in situ* biodegradation, its presence in the biotraps suggests a potential role in NAFC degradation in wetlands (49). We also found several bacterial classes involved in sulfate or sulfur reduction (*Desulfobulbia*, *Desulfovibriona*, *Desulfuromonadia*) that were more abundant in the planted mesocosms. Sulfate/sulfur reducers were also previously found in oil sands tailings (40), and *Desulfuromonadales* are potential naphthalene degraders (43).

*Clostridia*, *Dechloromonas,* and sulfate-reducers are generally found in anoxic environments, and they were not expected to be stimulated in the rhizosphere of our shallow mesocosms. In fact, we initially had expected to find aerobic NA degraders, like the ones mentioned in our recent review about CWTS for OSPW remediation (13). Surprisingly, no increases in typical NAFCs-degrading bacteria (e.g., *Pseudomonas, Acinetobacter, Geobacter,* etc.) were detected in *Carex*-planted mesocosms. Wetland plants are known to transfer oxygen to their roots through their parenchyma. However, most plant-transported oxygen is rapidly consumed by root respiration and microbial activity in their immediate vicinity (50). Evidence suggests that anaerobic metabolism remains dominant in constructed wetlands, particularly during active plant growth when most of the available oxygen is depleted within the root zone (51). This observation about “active” plants reconciles this study with our previous study, where most plant-associated bacteria were aerobic, probably because plant and microbial growth were weak and oxygen did not get consumed as rapidly (25). Another recent study on *Carex aquatilis* found that the presence of the plant did not cause measurable soil oxygenation (52), reinforcing the idea that anoxic microenvironments can persist within its rhizosphere when actively growing. These localized anoxic conditions likely provide a suitable niche for anaerobic bacteria, such as *Hungateiclostridiaceae*, *Dechloromonas*, and sulfate reducers.

The dominant class of NAFCs in the mesocosms was classical naphthenic acids (O_2_ class) (7). They made up more than 50% of the NAFCs throughout the time course, and the reduction in the total NAFC concentration was consequently mostly due to their degradation. The bacterial degradation of classical monocyclic naphthenic acid, such as cyclohexanecarboxylic acid (CHCA), proceeds through the benzoyl-CoA pathway, which is also the pathway used by bacteria for anaerobic degradation of aromatic hydrocarbons (53, 54). Other n-acyl cyclohexanes could follow a similar pathway after reduction of the side chains. For instance, cyclohexaneacetic acid (CHAA) was found to be an intermediate in the degradation of the longer chain cyclohexanebutyric acid (CHBA) (23). Interestingly, unpublished metatranscriptomic analyses of the mesocosm showed a heightened expression of phenylacetate (PAA) degradation genes in the rhizosphere of *Carex,* especially the gene encoding for the PAA-CoA ligase (paaK), which controls the first step of PAA degradation, the addition of CoA. We hypothesize that, like CHCA that gets partly aromatized to enter the benzoyl-CoA pathway, CHAA could be transformed to enter the PAA degradation pathway. Interestingly, PAA is a natural auxin (55), and genes related to its degradation are widely distributed (56). Degradation of PAA also proceeds through epoxidation of the aromatic ring rather than through double hydroxylation, which might be a way to conserve oxygen under limiting conditions (57). More work is needed to confirm which pathways are used by microorganisms to degrade naphthenic acids when associated with plants, both under oxic or anoxic conditions.

In the current experiment, extractable sulfur was shown to increase more than six times in the planted mesocosm sediments from the initial concentration, a trend that was not significant in the unplanted mesocosms (7), suggesting a heightened release of extractable S forms from the bound organic S contained in the sediments through microbial activities. Interestingly, many members of the *Clostridia* can use sulfur as a terminal electron acceptor, so it could very well be that the increased sulfur concentration in the rhizosphere increased the activities of sulfate- and sulfur-reducing bacteria (including our *Clostridia*). The other anaerobes found to increase in the rhizosphere of *Carex*, *Dechloromonas*, use nitrate as a terminal electron acceptor. Nitrate was below the detection limit in the sediments and for most of the water samples. Other than that, the lower relative abundance of the *Nitrospiraceae*—nitrite-oxidizer or commamox—in the planted rhizosphere would suggest a lower production of nitrate from nitrification. More studies linking terminal electron acceptors to CWTS efficiency could help optimize remediation of OSPW.

### Conclusion

When *Carex aquatilis* was present in our mesocosm, we observed an accelerated reduction in NAFCs, prompting us to look at the microbial community associated with the plants. We found many differences in the diversity and community composition, as well as the relative abundance of many taxa. One surprising result was that many of the microorganisms associated with the plants were anaerobes. Their presence and their potential functional capacity were coherent with the reduction of NAFCs. We are now working on isolating these microbes to confirm our findings. Once confirmed, it could change the paradigm behind the optimization of CWTS for OSPW remediation, with interventions aiming at optimizing the microbial activities in the absence of oxygen.

## MATERIALS AND METHODS

### Experimental design

**Fig 7:**
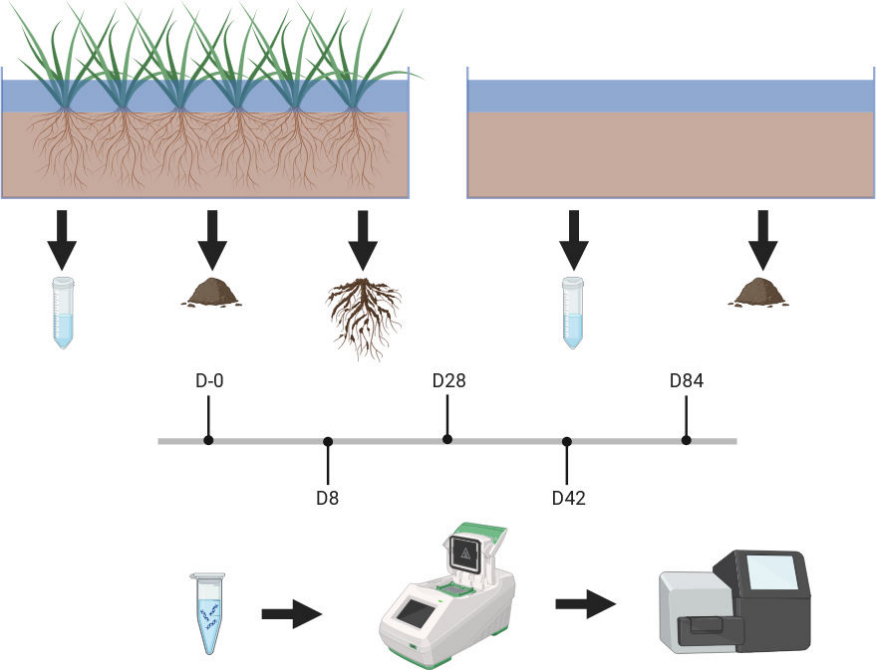
Experimental design. We sampled replicated planted (*Carex aquatilis*) and unplanted mesocosms at five time points (D-0, D8, D28, D42, and D84). We took water and sediments from the unplanted mesocosms, and water, sediments, roots, and rhizosphere sediments from the planted mesocosms. Created in https://BioRender.com.

The greenhouse mesocosm experiment was set up at the Northern Forestry Center in Edmonton, AB, Canada. The details of the experimental design have been provided in reference 7 and are summarized in Fig. 7. In summary, the experiment included two treatments, each replicated four times: (i) mesocosms containing 12 *Carex aquatilis* plants (three per mesocosm) with OSPW (Carex), and (ii) unplanted mesocosms containing only OSPW (OSPW). Each mesocosm was filled with coarse sand tailings and peat mineral mix and then topped with 25 cm (106.93 L) of OSPW. All material used in this experiment was sourced from Imperial’s Kearl oil sands mine. The mesocosms were operated as closed-loop, surface-flow systems, with OSPW pumped from a 159-L reservoir into each mesocosm at a flow rate of 20 mL/min, cycling approximately 28.8 L/day. The experiment ran for 84 days, for a total corresponding to 21 full cycles.

*Carex aquatilis* seeds were collected from the same ecoregion as the Imperial’s Kearl oil sands mine and grown in styroblock containers in greenhouse for 3 months. Once the plants reached an average height of 83 cm, they were transplanted into the mesocosms and acclimated with reverse osmosis (RO) water for a 32-day period. Following this, the mesocosms were flushed with a 50:50 mixture of OSPW and RO water for 7 days to gradually transition the plants to higher levels of OSPW. After flushing, the mesocosms were drained and refilled with 100% OSPW to initiate the experiment. The greenhouse conditions were maintained at a 16-h photoperiod with daytime temperature of 20°C and nighttime temperature of 10°C. To compensate for evaporation and maintain a consistent water volume, RO water was periodically added to the reservoir tanks.

### Sample collection

Samples for microbial community analyses were collected prior to 100% OSPW exposure, identified as D-0, and on days 8, 28, 42, and 84 after 100% OSPW exposure. Composite sediments and roots (which included rhizosphere) samples were collected from *Carex*-planted mesocosms, while only sediments were collected from the unplanted mesocosms. From the four mesocosms with *C. aquatilis,* two 50-mL conical tubes were filled with combined root and sediments collected at a depth of 0–30 cm from the sediment surface near a minimum of three plants per mesocosm. From the four unplanted mesocosms, two 50-mL conical tubes of sediment were collected from a minimum of three areas at a depth of 0–30 cm. The collected samples were then snap frozen and shipped on dry ice to Environmental Genomics laboratory of the Natural Resources Canada’s Laurentian Forestry Center where they were stored in a −80°C freezer until further processing. Water samples were collected using sterile Nalgene bottles and shipped on ice to the University of Calgary.

### Naphthenic acid fraction compound (NAFC) measurements

Water samples were extracted using an ENV+ solid-phase extraction (SPE) method (58). Briefly, sample aliquots were measured to ~100 mL (with exact volumes recorded) and acidified to pH < 2 with formic acid. The SPE cartridges were rinsed with 6 mL of Milli-Q water, 6 mL of LC-MS grade methanol (Fisher Scientific, Hampton NH, USA), and conditioned with a further 6 mL of Milli-Q water. Acidified samples were drawn through prepared cartridges at 3–5 mL/min under vacuum, rinsed with 6 mL of Milli-Q water to desalt, then dried under gentle vacuum. Sample extracts were eluted with 6 mL of LC-MS grade methanol and evaporated at 40°C under a gentle flow of N_2_ gas (5.0-grade; Linde Canada, Saskatoon, Saskatchewan). Dried sample elutions were reconstituted into 1 mL of 50:50 ACN:H_2_O with 0.1% NH_4_OH, then transferred to clean labeled 2.0-mL amber glass LC-MS vials. Sample extracts were analyzed using an LTQ Orbitrap Velos Elite mass spectrometer (Thermo Fisher Scientific, Waltham, MA) at 240,000 resolution (as measured at 400 m/z) operating in a negative-ion electrospray ionization mode, as previously described by reference 58.

### Genomic sample preparation

Water samples were filtered through a 0.2-μm PES membrane filter prior to DNA extraction. Sediment, rhizosphere, and root samples were thawed at 4°C for 72 h and poured into Petri dishes placed on ice. The roots were sorted while preserving the soil tightly attached to them (rhizosphere). Then, the remaining sediments were thoroughly homogenized, transferred into PowerBead Pro tubes (250 mg), provided with the DNeasy PowerSoil Pro kit (QIAGEN), frozen in liquid nitrogen, and stored at −80°C.

To collect the rhizosphere, fine roots were transferred into 50-mL Falcon tubes containing ~10–15 mL of PowerBead solution (QIAGEN). Tubes were shaken to dislodge the rhizosphere weakly attached to roots, placed in a bath sonicator (Branson 2210 ultrasonic cleaner; frequency of 40 kHz) for 30 s and centrifuged at 4°C, 4,500 rpm for 10 min. Two grams of roots was transferred into new 50-mL Falcon tubes containing fresh PowerBead solution, while the remaining pellets of rhizosphere were kept on ice. Three series of 30 s of sonication with a 30 s pause between each one were carried out for the 2 g-weighed roots. Roots were then transferred to Petri dishes placed on ice, with ~5–10 mL of fresh PowerBead solution, while the remaining pellets of rhizosphere and PowerBead solution were mixed with the corresponding pellets of rhizosphere previously kept on ice. Tubes containing rhizosphere were then centrifuged, according to the above-mentioned parameters, and supernatant was discarded. Using spatulas, recovered rhizosphere was thoroughly homogenized, transferred into PowerBead Pro tubes (250 mg), frozen in liquid nitrogen, and stored at −80°C.

Roots previously kept in Petri dishes placed on ice were cleared of debris using forceps and transferred into new 50-mL conical tubes, containing ~10 mL of fresh PowerBead solution. The sonication and centrifugation steps were repeated as above. Cleaned roots were pat dried and cut into small pieces. Up to 100 mg of roots was transferred into 2-mL Eppendorf Safe-Lock tubes, each containing one sterile 5-mm tungsten bead (QIAGEN), frozen in liquid nitrogen and stored at −80°C. Just prior to gDNA isolation, frozen roots were ground into a fine powder using the TissueLyser II (QIAGEN) at 26 Hz for ≥2 min 45 s, with snap-freezing  by immersing the tubes in liquid nitrogen between grinding cycles.

### DNA isolation, library preparation for metabarcoding, and sequencing

Isolation of DNA was performed with the DNeasy PowerSoil Pro kit. Two nanograms of exogenous DNA of *Thermus thermophilus* (strain HB8; 27634D-5, ATCC) was added as internal standard. The isolation was performed using QIAcube instruments (program *PowerSoil Pro, including IRT*; QIAGEN), and DNA recovery was evaluated using the dsDNA Broad Range assay kit (Invitrogen) and Qubit 3.0 fluorometer (Thermo Fisher Scientific). Solutions of DNA were diluted to 6 ng/µL within 10 mM Tris pH 8.0 solution, using the QIAgility automated system (QIAGEN).

The PCR amplification, library preparation for metabarcoding, and sequencing steps were performed at the Centre d’expertise et de services Génome Québec (Montréal, Canada). Bacterial and archaeal communities were targeted using the primer pair 515F-Y (59) and 926R (60), whereas the fungal community was targeted using the primer pair ITS9F (61) and ITS4R (62), which allow for the amplification of 16S rRNA gene and ITS2 region, respectively. Pooled libraries (in equivalent amount) were sequenced in paired-end format using the MiSeq Reagent Kit V3 600 cycles and the MiSeq instrument (Illumina). Both bacterial and fungal communities were sequenced for the solid samples (roots, rhizosphere, and sediments samples), while only bacterial communities were sequenced for the water column.

### Bioinformatics and statistical analysis

Bioinformatic analysis of sequence data was performed using Amplicon Tagger (63), as described in reference 64. All further data analyses on the resulting amplicon sequence variant (ASV) tables were processed using R language (R version 4.4.2) (65), and figures were generated with ggplot2 (66). For the ITS data set, we removed all the sequences that were not assigned as fungi. In the solid samples 16S data set, on the 5,057 ASVs found, 22 of them were classified as Archaea. As they encompassed only 0.15% ± 0.16% of the relative abundance in the samples, we removed these reads to only keep bacteria. The same procedure was done on the 16S water data set, which contained five Archaea ASVs on the 2,856 found and represented 0.006% ± 0.02% of the relative abundance. We measured three alpha diversity indices on the raw read counts of the different data sets: (i) observed richness, (ii) Shannon, and (iii) Simpson. Statistical differences between sample types or sampling dates were assessed using a post-hoc Dunn’s test with a Bonferroni *P*-value correction to account for multiple testing. We also calculated the alpha diversity indices on rarefied data and confirmed the results were similar to the ones obtained on raw data. To measure differences in microbial community composition between sample types, we used PERMANOVA tests on robust Aitchison (RCLR) distance matrices. This type of log ratio transformation is appropriate for the compositional nature of metabarcoding data (67). We restricted the permutations between sample types within each sampling date as microbial communities were significantly impacted by time. We used pairwise PERMANOVA as post-hoc tests when relevant but did not restrict permutations within sampling dates as we did not have enough statistical power. PERMANOVA tests were used with 999 permutations. Microbial community composition was assessed visually using principal coordinate analysis (PCoA). We used relative abundance of ASVs agglomerated at the taxonomic level of class (a compromise between taxonomic information and graphical clarity) to compare microbial community profile between sample types. We used DESeq2 (68) to detect differentially abundant taxa between sample types or time points. This analysis was performed on amplicon sequence variants (ASVs) agglomerated at the family or ASV level to allow for finer taxonomic resolution. Prior to the analysis, we removed the ASVs or families that were not present in at least 25% of the samples to reduce noise and increase the confidence in our results. We complemented this analysis using indicator species analysis (69). To analyze the relation of taxa of interest at the beginning of the experiment and the concentration of NAFCs at the end of our study, we measured correlations between the microbial abundance at the different sampling dates with the final NAFCs concentrations. The rationale was to find taxa that had an abundance negatively correlated with NAFCs concentration as they could potentially be involved in NAFCs degradation.

## Data Availability

Raw sequence data are available on the NCBI portal under accession identifier PRJNA1260004. The R scripts used to analyze the data and generate figures were deposited on the GitHub repository: https://github.com/SimonMorvan/Mesocosm1_Metabarcoding.

## SUPPLEMENTAL MATERIAL

Supplemental material aem.00410-26-s0001.pdf Figures S1 to S13.

ASM does not own the copyrights to Supplemental Material that may be linked to, or accessed through, an article. The authors have granted ASM a non-exclusive, world-wide license to publish the Supplemental Material files. Please contact the corresponding author directly for reuse.
