# Supplementary material for: Potential role of anaerobic plant-associated bacteria in naphthenic acid degradation
Source: Appl Environ Microbiol. 2026 Apr 27;92(5):e00410-26. doi: 10.1128/aem.00410-26 (PMC13188881; doi:10.1128/aem.00410-26)
Supplement: Supplemental material — Figures S1 to S13. [file aem.00410-26-s0001.pdf]

Potential role of anaerobic plant-associated bacteria in naphthenic acid degradation.

Simon Morvan[1], Sara Correa-García[1], Marie-Josée Bergeron[2], Kaitlyn Trepanier[3], Ian J. Vander Meulen[4,5], Dilini M. Atugala[7], Julien Tremblay[1], Jason M. E. Ahad[6], John V. Headley[4], Lisa M. Gieg[7], Dani Degenhardt[3], Christine Martineau[2], Étienne Yergeau[1*]

[1] Institut National de Recherche Scientifique, Laval, Canada

[2] Natural Resources Canada, Canadian Forest Service, Laurentian Forestry Centre, Québec, Canada

[3] Natural Resources Canada, Canadian Forest Service, Northern Forestry Centre, Edmonton, Canada

[4] Environment and Climate Change Canada, National Hydrology Research Centre, Saskatoon, Canada

[5] College of Engineering, Department of Civil, Geological and Environmental Engineering, University of Saskatchewan, Saskatoon, Canada

[6] Natural Resources Canada, Geological Survey of Canada, Québec, Canada

[7] Department of Biological Sciences, University of Calgary, Calgary, Canada

* Corresponding author: Étienne Yergeau, etienne.yergeau@inrs.ca

Supplementary figures

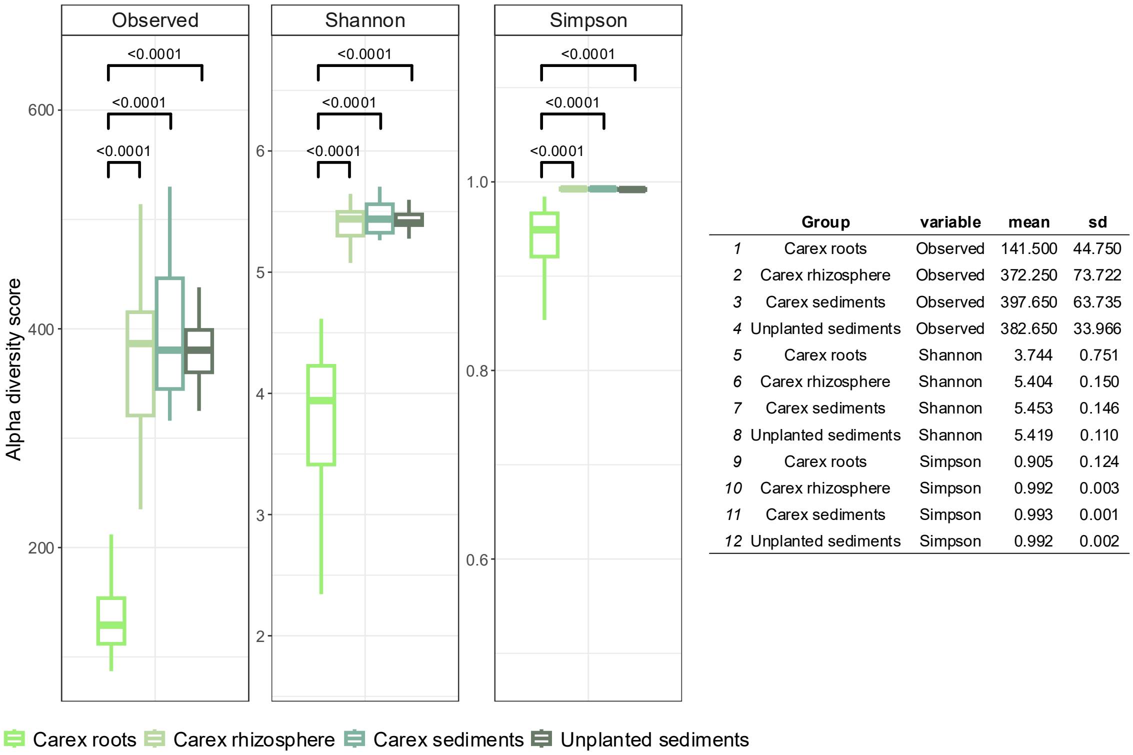

	Group	variable	mean	sd
1	Carex roots	Observed	141.500	44.750
2	Carex rhizosphere	Observed	372.250	73.722
3	Carex sediments	Observed	397.650	63.735
4	Unplanted sediments	Observed	382.650	33.966
5	Carex roots	Shannon	3.744	0.751
6	Carex rhizosphere	Shannon	5.404	0.150
7	Carex sediments	Shannon	5.453	0.146
8	Unplanted sediments	Shannon	5.419	0.110
9	Carex roots	Simpson	0.905	0.124
10	Carex rhizosphere	Simpson	0.992	0.003
11	Carex sediments	Simpson	0.993	0.001
12	Unplanted sediments	Simpson	0.992	0.002

Supplementary Figure 1 – Alpha diversity indices of the different sample types for the bacterial community.

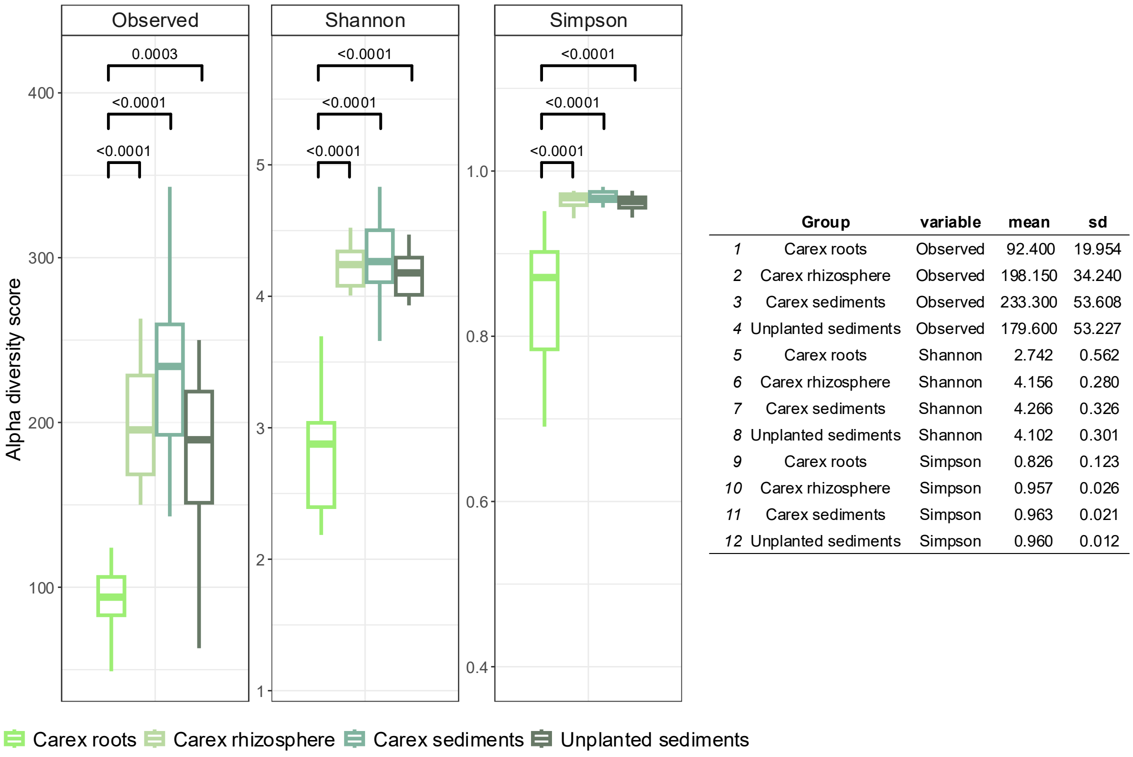

	Group	variable	mean	sd
1	Carex roots	Observed	92.400	19.954
2	Carex rhizosphere	Observed	198.150	34.240
3	Carex sediments	Observed	233.300	53.608
4	Unplanted sediments	Observed	179.600	53.227
5	Carex roots	Shannon	2.742	0.562
6	Carex rhizosphere	Shannon	4.156	0.280
7	Carex sediments	Shannon	4.266	0.326
8	Unplanted sediments	Shannon	4.102	0.301
9	Carex roots	Simpson	0.826	0.123
10	Carex rhizosphere	Simpson	0.957	0.026
11	Carex sediments	Simpson	0.963	0.021
12	Unplanted sediments	Simpson	0.960	0.012

Supplementary Figure 2 - Alpha diversity indices of the different sample types for the fungal community.

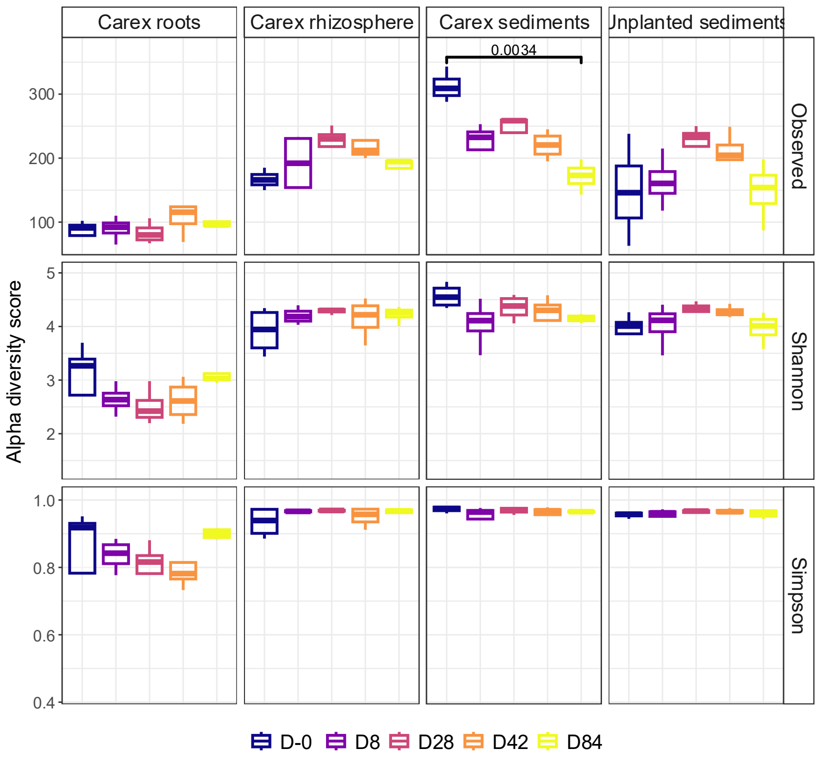

	Time	variable	mean	sd
1	D-0	Observed	177.625	94.032
2	D8	Observed	166.875	60.264
3	D28	Observed	193.312	70.682
4	D42	Observed	190.375	55.270
5	D84	Observed	151.125	42.066
6	D-0	Shannon	3.815	0.864
7	D8	Shannon	3.729	0.718
8	D28	Shannon	3.865	0.836
9	D42	Shannon	3.814	0.777
10	D84	Shannon	3.859	0.495
11	D-0	Simpson	0.915	0.142
12	D8	Simpson	0.927	0.062
13	D28	Simpson	0.925	0.083
14	D42	Simpson	0.919	0.080
15	D84	Simpson	0.947	0.030

Supplementary Figure 3 - Time effect on alpha diversity indices of the different sample types for the fungal community.

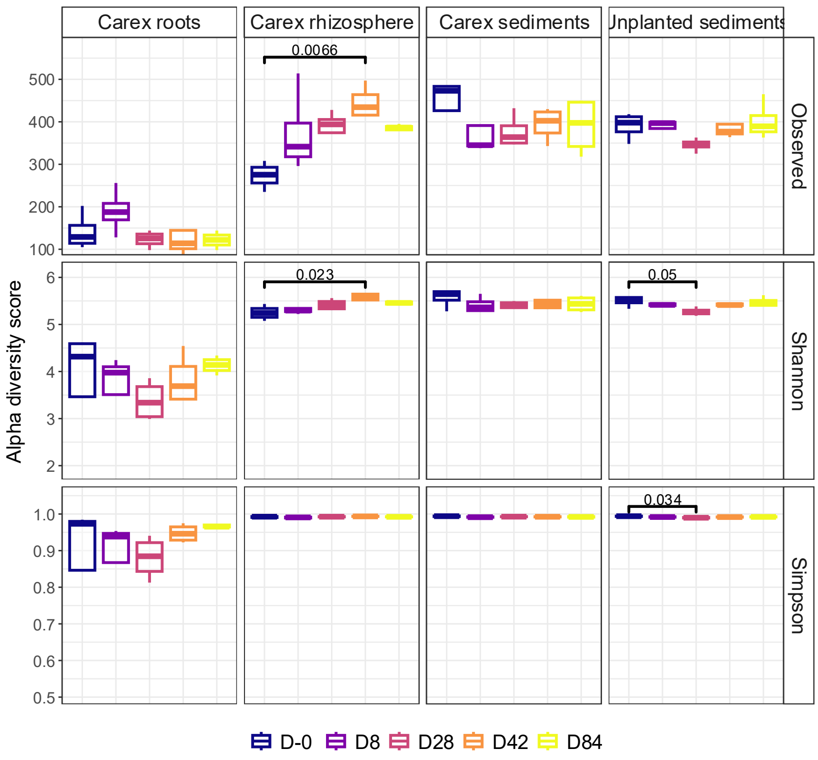

	Time	variable	mean	sd
1	D-0	Observed	310.500	126.675
2	D8	Observed	334.688	108.673
3	D28	Observed	307.875	114.674
4	D42	Observed	340.125	131.918
5	D84	Observed	324.375	126.803
6	D-0	Shannon	5.014	0.994
7	D8	Shannon	4.948	0.881
8	D28	Shannon	4.871	0.913
9	D42	Shannon	5.067	0.781
10	D84	Shannon	5.125	0.603
11	D-0	Simpson	0.958	0.127
12	D8	Simpson	0.962	0.079
13	D28	Simpson	0.964	0.056
14	D42	Simpson	0.981	0.023
15	D84	Simpson	0.986	0.011

Supplementary Figure 4 – Time effect on alpha diversity indices of the different sample types for the bacterial community.

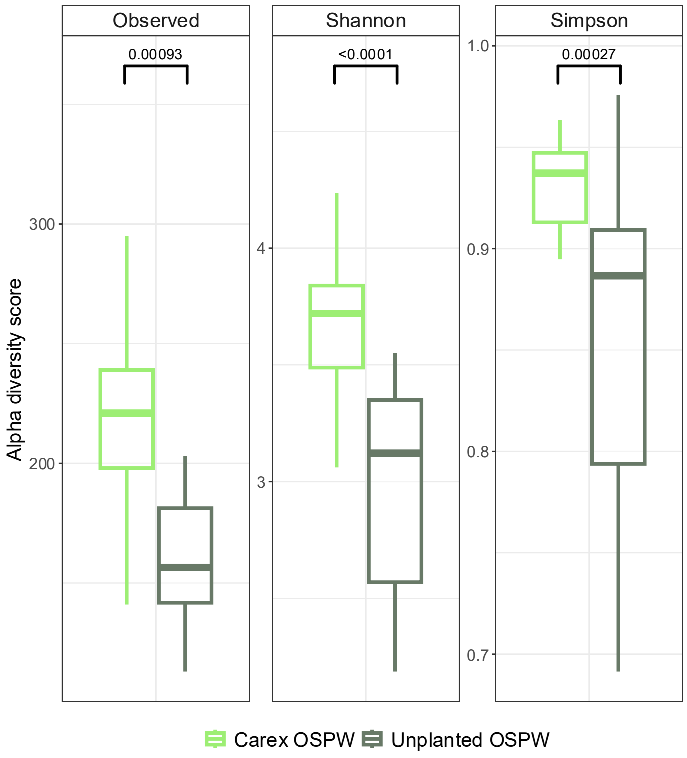

	Group	variable	mean	sd
1	Carex OSPW	Observed	219.316	42.732
2	Unplanted OSPW	Observed	172.556	59.460
3	Carex OSPW	Shannon	3.687	0.298
4	Unplanted OSPW	Shannon	3.041	0.604
5	Carex OSPW	Simpson	0.933	0.021
6	Unplanted OSPW	Simpson	0.858	0.079

Supplementary Figure 5 - Alpha diversity indices of the different sample types for the water bacterial community.

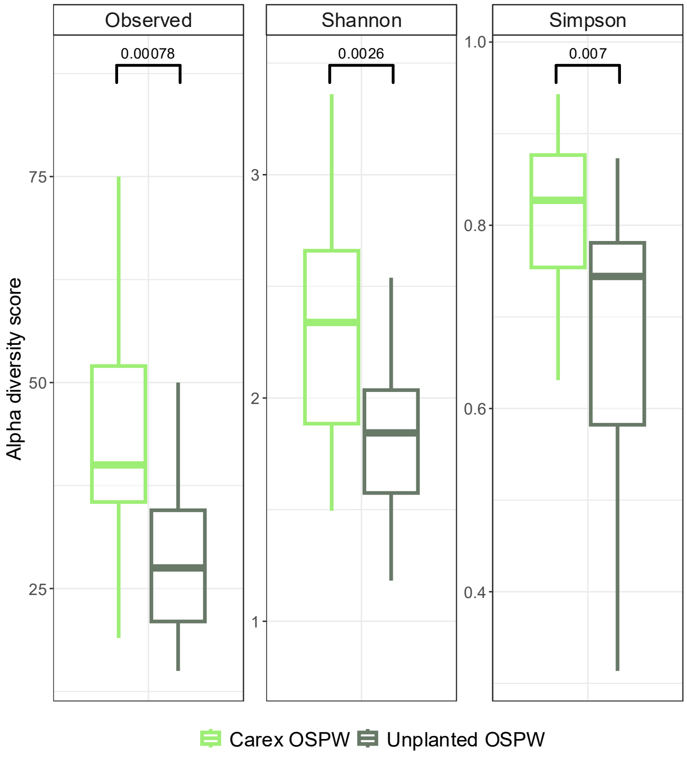

	Group	variable	mean	sd
1	Carex OSPW	Observed	43.474	16.467
2	Unplanted OSPW	Observed	28.000	9.353
3	Carex OSPW	Shannon	2.360	0.537
4	Unplanted OSPW	Shannon	1.798	0.420
5	Carex OSPW	Simpson	0.805	0.107
6	Unplanted OSPW	Simpson	0.690	0.143

Supplementary Figure 6 - Alpha diversity indices of the different sample types for the water eukaryote community.

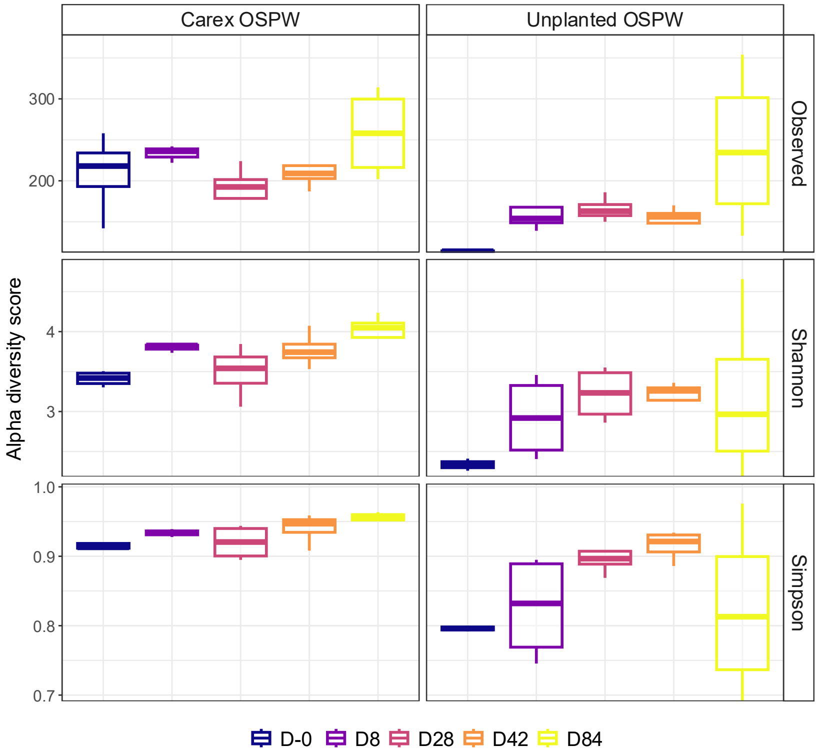

	Time	variable	mean	sd
1	D-0	Observed	177.333	61.992
2	D8	Observed	192.857	43.122
3	D28	Observed	176.500	27.276
4	D42	Observed	182.375	37.305
5	D84	Observed	248.500	74.770
6	D-0	Shannon	3.053	0.563
7	D8	Shannon	3.299	0.596
8	D28	Shannon	3.358	0.344
9	D42	Shannon	3.476	0.381
10	D84	Shannon	3.589	0.843
11	D-0	Simpson	0.877	0.063
12	D8	Simpson	0.872	0.079
13	D28	Simpson	0.910	0.027
14	D42	Simpson	0.928	0.024
15	D84	Simpson	0.889	0.109

Supplementary Figure 7 - Time effect on alpha diversity indices of the different sample types for the water bacterial community.

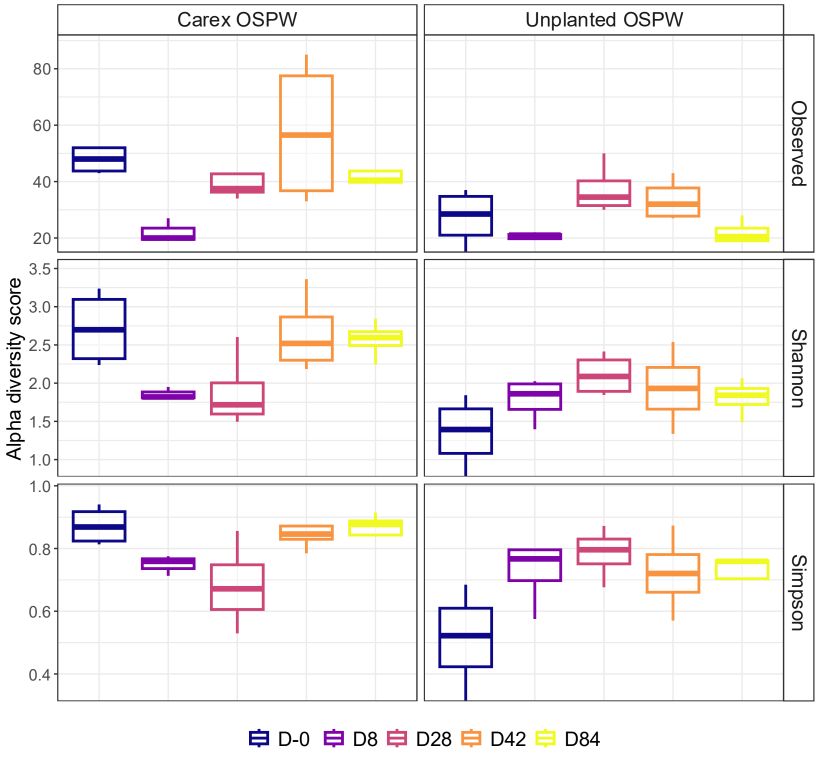

	Time	variable	mean	sd
1	D-0	Observed	37.500	13.213
2	D8	Observed	20.857	3.338
3	D28	Observed	39.375	9.319
4	D42	Observed	45.625	22.000
5	D84	Observed	32.500	12.224
6	D-0	Shannon	2.035	0.857
7	D8	Shannon	1.816	0.212
8	D28	Shannon	1.996	0.391
9	D42	Shannon	2.290	0.611
10	D84	Shannon	2.189	0.466
11	D-0	Simpson	0.692	0.224
12	D8	Simpson	0.736	0.077
13	D28	Simpson	0.734	0.119
14	D42	Simpson	0.788	0.117
15	D84	Simpson	0.780	0.118

Supplementary Figure 8 – Time effect on alpha diversity indices of the different sample types for the water eukaryote community.

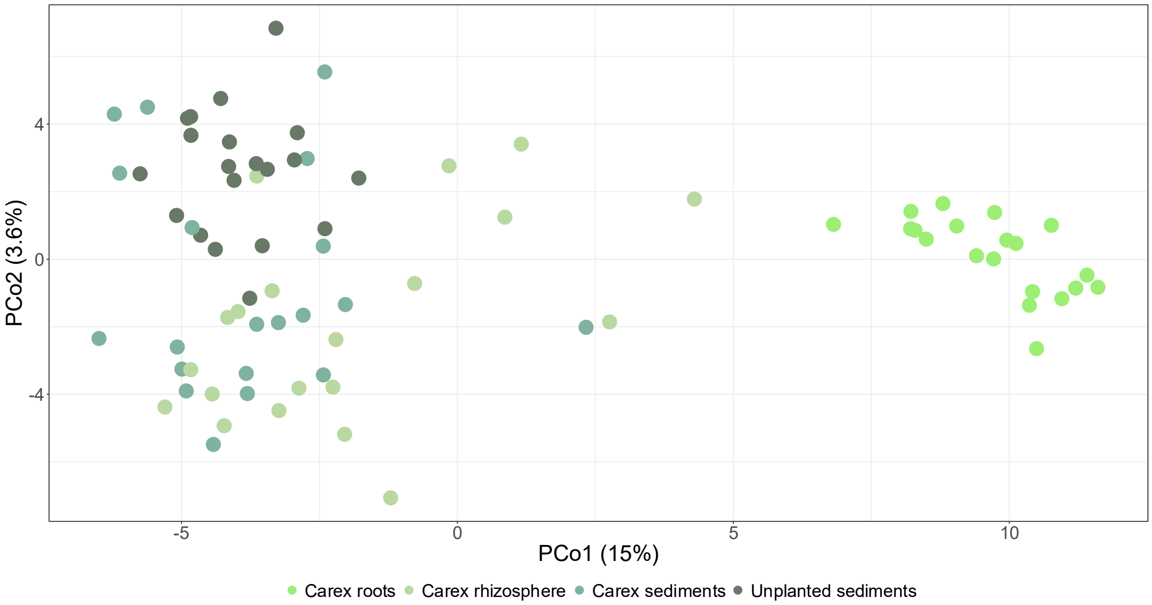

Supplementary Figure 9 – Ordination presenting the beta diversity of the bacterial communities based on robust Aitchison distance matrices and principal coordinate analysis (PCoA).

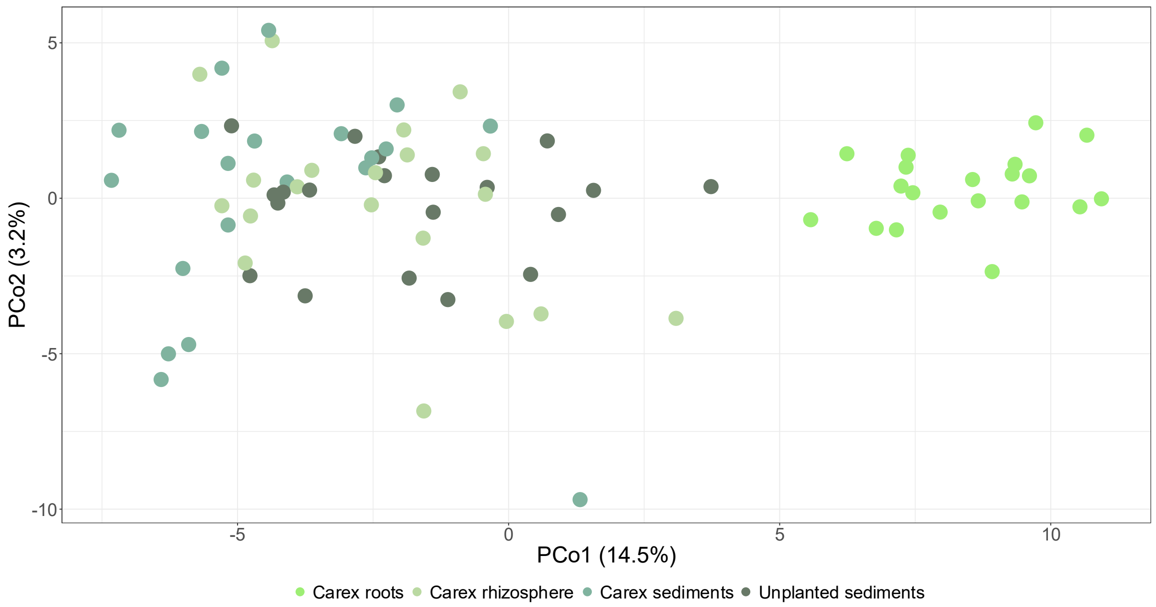

Supplementary Figure 10 - Ordination presenting the beta diversity of the fungal communities based on robust Aitchison distance matrices and principal coordinate analysis (PCoA).

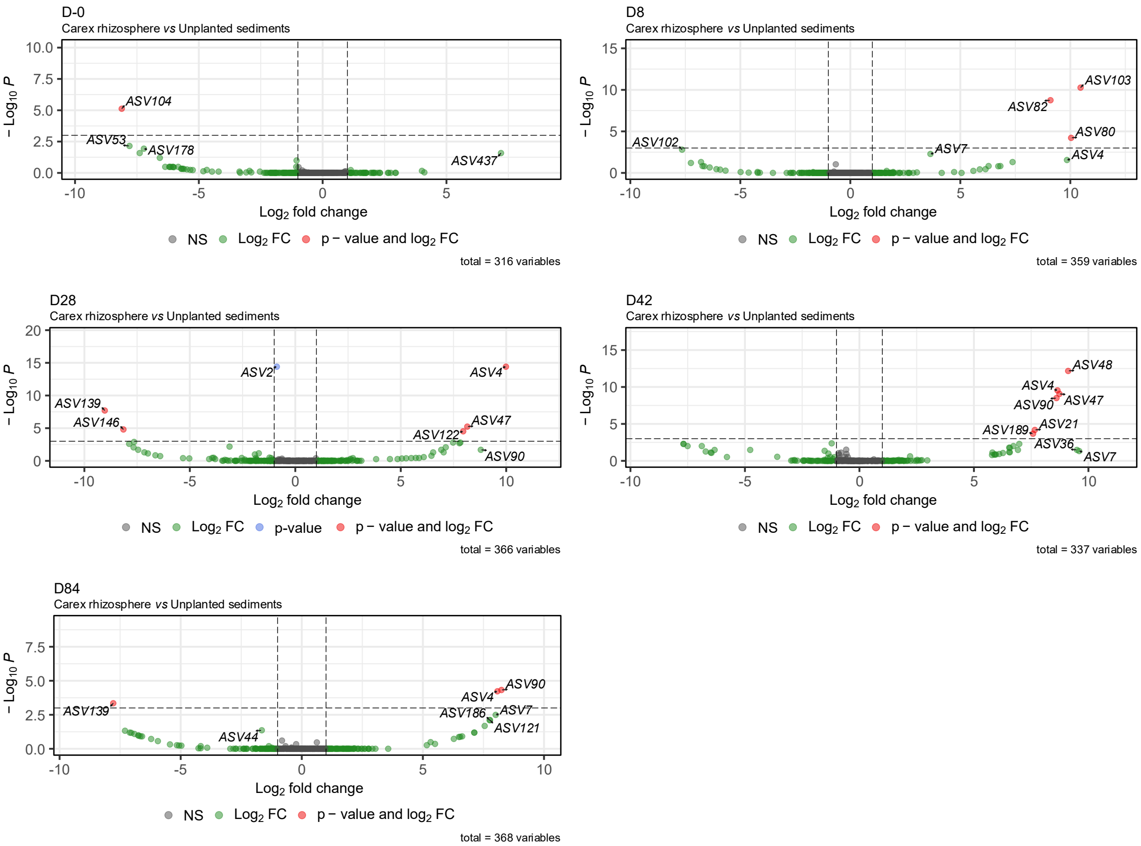

Supplementary Figure 11 – Differential abundance in bacterial ASVs between Carex rhizosphere and OSPW unplanted sediments for each time point.

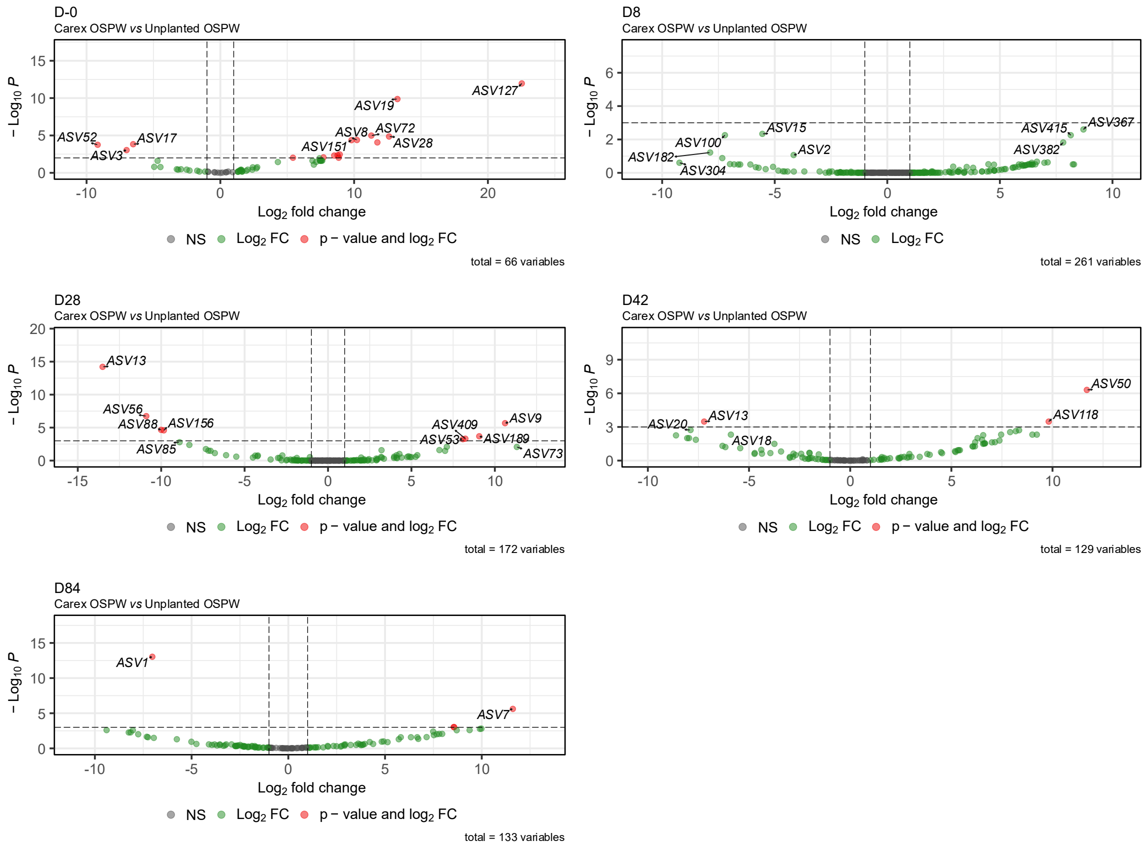

Supplementary Figure 12 – Differential abundance between Carex water and OSPW water for each time point.

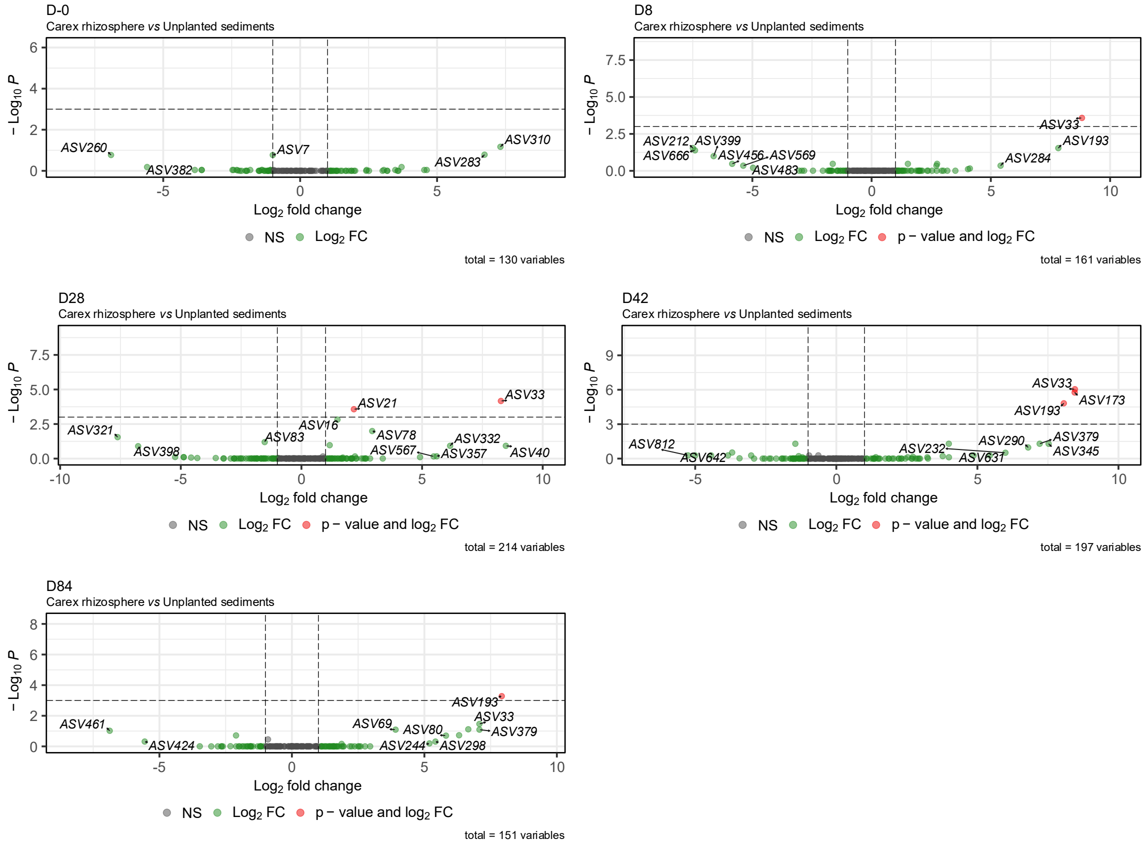

Supplementary Figure 13 – Differential abundance in fungal ASVs between Carex rhizosphere and OSPW unplanted sediments for each time point.
